# User experiences of an app-based mHealth intervention (MINISTOP 2.0) integrated in Swedish primary child healthcare among Swedish-, Somali- and Arabic-speaking parents and child healthcare nurses: A qualitative study

**DOI:** 10.1177/20552076231203630

**Published:** 2023-09-24

**Authors:** Christina Alexandrou, Stina Rutberg, Linnea Johansson, Anna-Karin Lindqvist, Ulrika Müssener, Marie Löf

**Affiliations:** 1 Department of Health, Medicine and Caring Sciences, Division of Society and Health, 4566 Linköping University, Linköping, Sweden; 2 Department of Biosciences and Nutrition, 27106 Karolinska Institutet, NEO, Group MLÖ, Huddinge, Sweden; 3 Department of Health Sciences, Division of Health, Medicine and Rehabilitation, Luleå University of Technology, Luleå, Sweden

**Keywords:** Parental support, mHealth, app-based, preschool, healthy lifestyle behaviors, child healthcare

## Abstract

**Background:**

Preventive and scalable interventions, accessible to all, to counteract childhood obesity are urgently needed. We have recently developed a novel, digital parental intervention (MINISTOP 2.0 app) available in Swedish, Somali, Arabic and English. We have previously reported its positive effects on children's health behaviors and on parental self-efficacy. However, before introducing the app at scale in primary child healthcare, implementation aspects also need to be explored.

**Aim:**

This study aims to explore and describe user experiences as well as acceptability and feasibility of the MINISTOP 2.0 app-based intervention in a diverse group of parents (end-users) and Swedish child healthcare nurses (implementers).

**Methods:**

Individual interviews were conducted with Swedish- (*n* = 9), Somali- (*n* = 9), Arabic- (*n* = 5) and English-speaking (*n* = 1) parents as well as Swedish primary child healthcare nurses (*n* = 15). Data was analyzed using content analysis with an inductive latent approach.

**Results:**

Parents described how the app facilitated behavior change through increased awareness regarding current diet and physical activity behaviors. Furthermore, the evidence-based app content further facilitated trust and behavior change. Both parents and nurses acknowledged the app's preventive potential and the potential for reaching parents with diverse backgrounds or in need of extra support.

**Conclusion:**

The MINISTOP 2.0 app was perceived as a useful tool for health promotion both by parents and healthcare professionals, especially since it was adapted to several languages. These findings coupled with the previously shown beneficial effects on health behaviors support the large-scale implementation of the app in primary child healthcare.

## Background

Childhood overweight and obesity is a global health challenge.^1,2^ The development of overweight and obesity early in life often tracks into adulthood where these conditions are associated with increased risk of cardiometabolic disease as well as impaired psychosocial health^3–7^. Childhood overweight and obesity is also linked to health inequities, with a much higher prevalence in socioeconomically vulnerable and migrant populations already in the preschool age^8–10^. Indeed, it is relevant to note that ages 0–5 years have been suggested as critical for preventing development of overweight and obesity.^11^ This calls for preventive efforts and interventions that reach and are accessible for all parents during the preschool years.^12^ Mobile health (mHealth) interventions are of high public health relevance^13–15^ as they enable delivery of evidence-based preventive interventions at scale, through healthcare systems, and also provide opportunity for increased reach of diverse populations.

**Figure 1.**
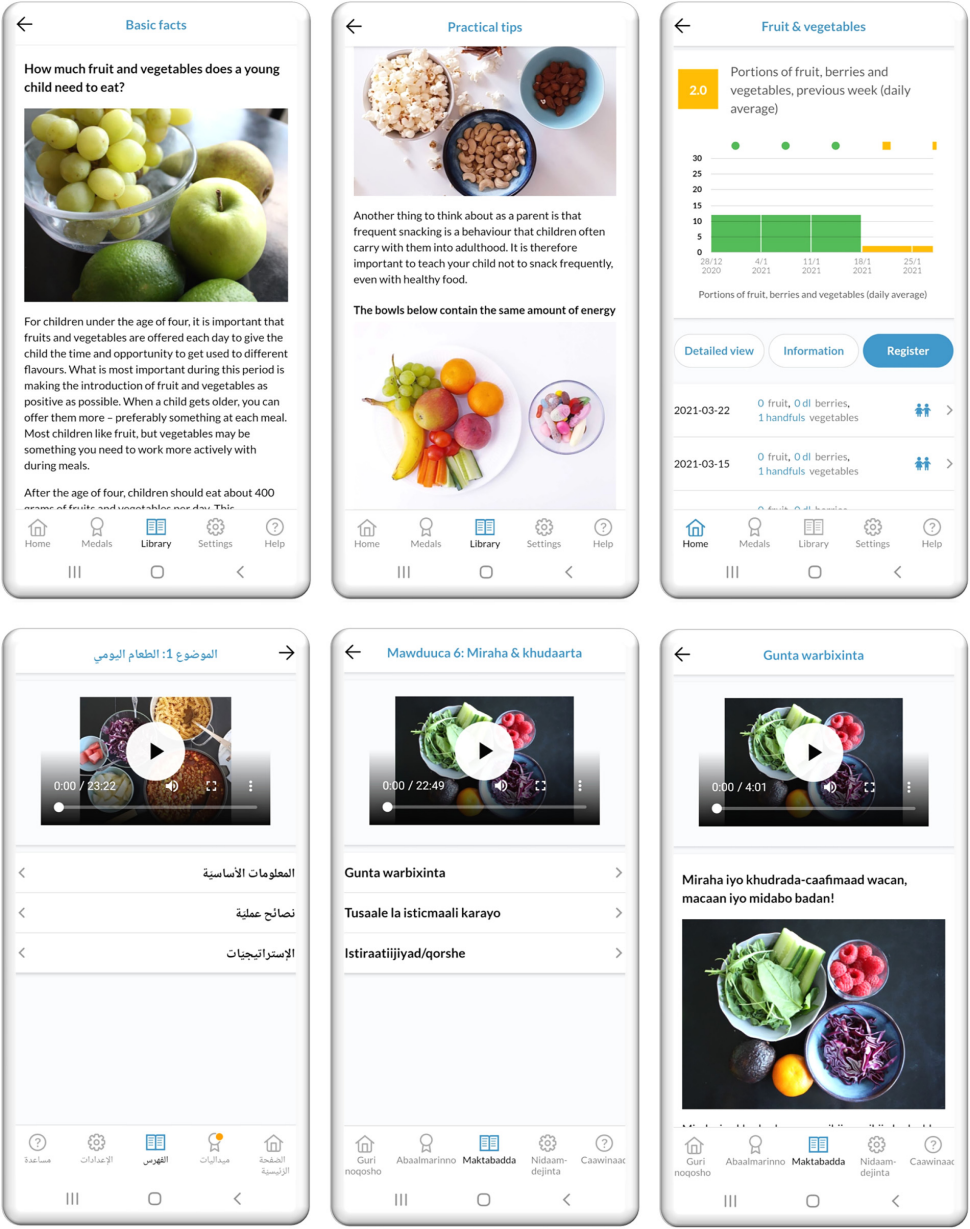
Screenshots from the MINISTOP 2.0 app. The first row displays examples from the information and the registration feature in the app. The second row shows examples of audio/video files of the information content in the Somali and Arabic language version of the app. This audio/video feature was added to facilitate accessibility of the content in the app for parents with low literacy. The MINISTOP 2.0 app is currently available in Swedish, Somali, Arabic and English.

We have recently developed a novel mHealth intervention (MINISTOP), aiming to promote healthier eating and physical activity in 2.5/-3-year-olds.^16–18^ The MINISTOP intervention is a 6-month-long app-based support program for parents, informed by social cognitive theory^19^ and various behavior change techniques^20^ such as providing general information, action planning, self-monitoring of behavior and feedback on behavior. The MINISTOP app (Figure 1) delivers information, practical tips and strategies to guide parents in creating healthy food environments and how to be role models for healthy diet and physical activity behaviors. Furthermore, the app focuses on helping parents understand the importance of learning their child's hunger and satiety cues and allowing their child to become more autonomous in their eating. Taken together, these strategies^21,22^ facilitate learning of self-regulation of appetite in the child that, in the long-term, may have a protective effect against the development of overweight and obesity. In order to facilitate accessibility of the information for parents with low literacy, the text in the app is also available as audio/video files (Figure 1). Additionally, the intervention includes strategies on how to set healthy boundaries for intakes of unhealthy foods and for screen time. The app also contains a registration feature for self-monitoring of children's dietary and physical activity behaviors.^17^ Parents are thus able to register their child's daily intakes of fruit and vegetables, sweet and savory treats and sweet drinks, as well as time spent being physically active and in front of screens. At the end of each week, parents receive feedback (graphical and as a push notification) based on their registrations.

The MINISTOP intervention has undergone rigorous scientific evaluation and has shown positive results on children's lifestyle behaviors, both in an efficacy trial in 2014–2015 (MINISTOP 1.0, *n* = 315)^16^ and in a real-world effectiveness trial within Swedish primary child healthcare 2019–2022 (MINISTOP 2.0, *n* = 552).^17,18^ In the MINISTOP 2.0 effectiveness trial,^18^ we managed to recruit a study population where 24% of the children had two foreign-born parents, a percentage that reflects the general Swedish population.^23^ This was primarily achieved by conducting the study within primary child healthcare. In Sweden, child healthcare is free of charge, and all children aged 0–5 years are invited to attend regular visits for health check-ups and developmental screening tests.^24,25^ Furthermore, prior to study start, the app was also adapted and translated into Somali, Arabic and English^26^ as these are the most common languages currently spoken in Sweden after Swedish.^27^ The main results of the effectiveness evaluation of the MINISTOP 2.0 app showed statistically significant beneficial effects on parent-reported health behaviors in children (i.e. decreased intakes of sweet and savory treats, sweet drinks and screen time) as well as improved parental self-efficacy (PSE).^18^

Noteworthy, the MINISTOP 2.0 study^17,18^ used an overall hybrid type 1 effectiveness–implementation design^28^ to also enable exploration of implementation aspects such as intervention acceptability and feasibility,^29^ as intervention effectiveness alone is not a sole predictor of whether an intervention will be successfully implemented or not.^29^ Investigating such implementation aspects is important as they help optimize and guide implementation processes within their intended settings.^29^ In this paper, we report the main results from the implementation outcomes of the MINISTOP 2.0 hybrid type 1 study. Specifically, the present study aims to explore and describe user experiences as well as acceptability and feasibility of the MINISTOP 2.0 app-based intervention in a diverse group of parents (end-users) and Swedish child healthcare nurses (implementers). The parents included were either Swedish-, Somali-, Arabic- or English-speaking.

## Methods

### Study design

This study used a qualitative study design^30^ conducted through individual interviews. The Consolidated Criteria for Reporting Qualitative Research (COREQ) checklist was used.^31^

### Participants and recruitment

Purposive sampling was used for the recruitment of both parents (*n* = 24) and child healthcare nurses (*n* = 15). Parents were found eligible for participation if they had used the MINISTOP 2.0 app^17,18^ and were willing to share their user experiences; however, there was no specific requirement regarding the amount they used the app. For nurses, inclusion criteria were participation in the recruitment of participants in the MINISTOP 2.0 trial and have experience using the MINISTOP 2.0 caregiver interface.

#### Parents

Parents from the intervention group that spoke Swedish and/or English were recruited in conjunction with an evaluation questionnaire sent out by email at the end of the 6-month intervention period. In total, nine Swedish-speaking parents and one English-speaking parent indicated interest and thus were emailed additional written study and consent information prior to scheduling an appointment for a phone interview. To ensure that the study purpose and consent information was fully understood, Somali- and Arabic-speaking parents that were not fluent in Swedish or English were approached and recruited by health communicators, that is, individuals linked to the healthcare system to facilitate communication with individuals with their specific cultural background. Overall, 41 Somali- and Arabic-speaking parents received information about the interview, and 9 Somali- and 5 Arabic-speaking parents consented to participate.

#### Nurses

An invitation with enclosed study and consent information was emailed to the nurses (*n* = 62) that had participated in the recruitment and data collection of the MINISTOP 2.0 trial. The invitation also included information regarding voluntary participation and the right to withdraw at any timepoint. An appointment for an individual phone interview was then scheduled with the first 15 nurses that replied, as they represented diverse geographic and socioeconomic contexts.

### Data collection

To capture variations in informant experiences, semi-structured interview guides (Supplementary file S1) were developed within the research group, with expertise in nutrition, physiotherapy, behavioral science and mHealth interventions. The interview guides included both specific questions regarding the features and content of the app (parents and nurses) and caregiver interface (nurses) alongside more wide and open questions regarding the informants’ overall user experience in relation to their current health behaviors (parents) and daily practice (nurses). Probing questions were used to clarify and to help the informants to further elaborate their answers. All interviews were audio recorded and transcribed verbatim by an external transcribing firm.

#### Parents

Parents were recruited between September 2020 and March 2022. Interviews were conducted over the phone by C.A., a female nutritionist and PhD student responsible for coordinating the recruitment and data collection in the MINISTOP 2.0 trial. C.A. had no previous relationship with the parents as the recruitment and data collection in the trial was conducted by the child healthcare nurses. Informed written consent was obtained from all informants before enrollment in the study. Additionally, verbal consent was recorded at the beginning of each interview. At the time of the interview, parents also answered demographic questions about themselves, including age, country of birth, educational attainment, occupation as well as number and age of their children. The interviewed parents (*n* = 24; 23 mothers, 1 father) were Swedish- (*n* = 9), Somali- (*n* = 9), Arabic- (*n* = 5) or English-speaking (*n* = 1). All Somali-speaking parents (*n* = 9) were born in Somalia, while the Arabic-speaking parents (*n* = 5) were born in different countries (Egypt, Iraq, Lebanon, Morocco and Syria). Level of education ranged from 2 up to 22 years (mean: 12.5 years). Parents were between 22 and 53 years of age (mean: 36 years), and the number of children they had ranged from one to seven (mean: three children). When needed, interviews with Somali- and Arabic-speaking parents were conducted together with a translator. Interviews were on average 48 minutes long and lasted between 32 and 74 minutes. Interviews conducted together with a translator (*n* = 7) were in general longer in duration.

#### Nurses

Nurses were recruited between May 2021 and January 2022, and interviews were conducted by C.A. and another PhD student in the research group—M.F., a female physiotherapist with expertise in implementation research. The nurses had no previous relationship with M.F.; however, due to the coordinating nature of her role in the trial, C.A. had met the nurses earlier when introducing the intervention. At the time of each interview, information about the nurses’ age, professional expertise and years of working experience was collected. All nurses (*n* = 15) were female; they were between 34 and 58 years of age (mean: 43.7 years), and their years of working experience within the profession ranged from 1 to 12 years (mean: 7.1 years). Informed verbal consent was recorded at the start of the interview. Interviews lasted on average 49 minutes and ranged between 27 and 67 minutes.

### Data analysis

The data collected contained rich information and was judged, after comprehensive discussions between the authors, to have reached saturation and thus as adequate to meet the aims of the study. Content analysis with an inductive latent approach inspired by Graneheim and Lundman^32^ was used to analyze, explore and acquire a deeper understanding of the data. To obtain an overview of the information in the interviews, all transcripts were fully read by C.A. (parents and nurses) and L.J. (nurses only), a female researcher and physiotherapist. A coding process guided by the aim of the study was then initiated separately by C.A. (parents and nurses) and L.J. (nurses). Data was divided into meaning units that were condensed into smaller meaning units and then further abstracted into codes. C.A. and L.J. reviewed each other's codes, and discrepancies were discussed to reach consensus and to ensure that the interpretation of data was correct. The codes were additionally reviewed by S.R., a female researcher and physiotherapist with expertise in qualitative methodology, who also supported the analysis process through regular discussions with C.A. These discussions also facilitated reflexivity by identifying and challenging the authors’ pre-understanding of the topic. When agreement around the coding datasets was reached, C.A. sorted the condensed meaning units and codes into preliminary sub-categories that were close to the text. Categories for all the data were then identified, by reading and re-reading the condensed meaning units, codes and preliminary categories several times. Finally, themes and sub-themes were identified through interpretation of the underlying meaning of the categories. The theme content was then jointly discussed with S.R., L.J. and A-K.L., a female researcher and physiotherapist, until final consensus was reached.

### Ethical considerations

This qualitative study was approved by the Swedish Ethical Review Authority (Ref No. 2019-02747; 21 August 2019).

## Results

**Figure 2.**
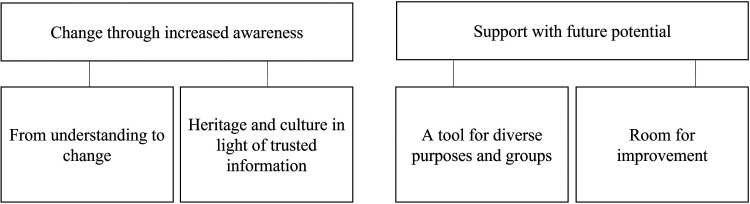
Overview of the themes and sub-themes.

The present study explored the overall user experiences and feasibility of the MINISTOP 2.0 app among parents and child healthcare nurses. Two themes, based on two sub-themes each, were identified in the analyses (Figure 2). Excerpts from the interviews were included to support the theme categorization. For the parents, quotations were denoted with sex, age and spoken language(s), as well as with information on whether an interpreter had been used during the interview. For the nurses, quotations were denoted with age and number of years in the profession.

### Change through increased awareness

The app facilitated behavior change among parents through increased awareness, and depending on previous experiences and current health behaviors, the app also enabled insights into habits grounded in cultural background and heritage. Furthermore, having support from an evidence-based tool was valued by both parents and nurses and facilitated both trust and behavior change.

#### From understanding to change

A red thread throughout the interviews was the parents’ perceptions of insight in relation to their family's current diet and physical activity behaviors after using the app. The app contributed to increased knowledge and insight that led to many small changes in everyday behaviors, such as learning to identify healthier foods in the supermarket, increasing the intake of vegetables during meals, drinking water instead of sweetened drinks, decreasing the intake of sweet treats as well as keeping a more mindful disposition of time spent in front of screens and in active play during the day. Although some parents perceived themselves as already knowledgeable, using the app was still described as worthwhile, as it brought both new inspiration and useful repetition. Others expressed how the app had provided insight on dietary behaviors previously established in their family, not being aware that these were unhealthy.

So, I used to give him different kinds of treats during the day, and I used to feel that it’s okay. But after using this app, it helped me understand some things that I used to do were not good for his health. Like giving him biscuits every day … because biscuits contain sugar, and that is not okay. (Arabic/English-speaking mother, 28 years)

Visualization of lifestyle behaviors was expressed as both valuable and necessary to improve habits, and parents shared how the registration feature increased awareness of what their children ate by making it more concrete. Specifically, registration in combination with supporting information provided an opportunity to work with and adjust current behaviors. For some, registration provided more structure to everyday life; however, daily or long-term registration was not necessary for creating positive insights or new routines. Reaching the recommendation by the end of the week was motivating and provided a sense of accomplishment. Even so, not reaching the recommendations was sometimes also perceived as helpful, as it led to increased awareness of the behavior and subsequent change. Likewise, registration of “bad days” provided insight and was described as a strong motivator to do better.

It was a bit painful, but in a good way I think, when it turned red [goal not reached]. It made you think ‘yes, but now it's time to get it together.’ (Swedish-speaking mother, 39 years)

Furthermore, the tone in the feedback messages, where positive reinforcement was used instead of negative feedback on health consequences, was also described to facilitate behavior change. Nevertheless, the registration feature was not appreciated by everyone; some had mixed feelings but thought it was good that the opportunity existed. However, others perceived it as stressful or unnecessary for behavior change and felt more affected by the information in the app.

Using the information feature was described as developing; it provided a good foundation of knowledge that led to new routines around food intake and eating behaviors as well as a sense of having control. The app was also appreciated for its holistic approach, where it both increased knowledge and awareness on health behaviors through general information and provided tips and strategies on how to set healthy boundaries and handle challenging situations. Moreover, parents appreciated the comprehensiveness of the app; that it gathered relevant information about health behaviors for preschool-aged children that otherwise would require visiting many different webpages. This also facilitated learning and understanding. Access to trustworthy information in one place was also appreciated by the nurses, who brought up how common it was for parents to search for information online, sometimes ending up on pages with questionable content.

Overall, it felt like the app was very thought through. There was not only [information] about ‘this is good to eat’, but you also got suggestions … if the child was not interested [in food, vegetables etc.], how to proceed and so on. (Swedish-speaking mother, 37 years)

Although parents in general had a good understanding of healthy and unhealthy foods, parents expressed a need for relevant information and examples regarding age-appropriate portion sizes, as well as healthy quantities of sweet and savory treats and sweet drinks. Thus, for some, information about learning, understanding and trusting the child's hunger and satiety cues in combination with pictures showing examples of age-appropriate portions increased awareness; for others, the information confirmed that the child was eating enough and helped reduce worries when eating meals perceived as too small by the parent. Moreover, strategies for healthy eating behaviors, such as starting with a smaller portion instead of serving a large portion at once, was also expressed as valuable knowledge.

For example, in the mornings, before I had used this app, my daughter had a hard time eating in the morning. When she didn’t eat anything [breakfast] before preschool, I used to worry and think ‘maybe she’s really hungry.’ But when I read in the app that if the child is alert and playing, then it’s fine. (Somali-speaking mother, 35 years)

Depending on previous knowledge, the physical activity content in the app also influenced parents on different levels. For instance, not everyone was aware of the recommendation on physical activity for 2-to-3-year-old children and described this information as helpful, as it made them more mindful about the issue. Others were inspired by the age-appropriate tips on active play for children and appreciated the tips on how to be more active together with their child.

I also thought that [the information on] active play was very good. That you got a little bit about ‘what is active play’? And ‘what counts as active play’? After all, you want your child to be active and healthy. And here, you got something to relate to. (Swedish-speaking mother, 36 years)

Furthermore, the tips on active indoor play were described as especially inspiring and novel. Through information and video examples, the app helped normalize active indoor play and shift the parents’ level of tolerance regarding this. Additionally, parents described how they consciously began allowing and enabling more active indoor play because of this.

#### Heritage and culture in light of trusted information

The app included features to reach, inform and inspire parents, and depending on heritage and cultural background, the app influenced awareness on different levels. For some of the parents, insights acquired from the information in the app generated discussions on the importance of receiving information from a trusted source to be able to identify sub-optimal health behaviors that were common in their culture but also to keep the habits that were good. As parents often relied on information from within their own social or cultural circle, that is, from individuals with the same way of life and similar food and eating behaviors as themselves, information from a trusted source was described as a prerequisite for questioning one’s habits and heritage. Parents also expressed how they had gained increased awareness regarding common practices for healthy diet and eating behaviors in their culture compared to research-based recommendations after using the app and highlighted how this was both motivating and reassuring when making lifestyle changes.

Before [using the app], you didn’t have much knowledge or experience, and you felt quite new. But after watching the videos, you feel like you have knowledge to base your actions on. And that’s more motivating … that your actions are now based on knowledge and facts. (Somali-speaking parent, 36 years (with interpreter))

Nurses also expressed a need to bridge and be able to communicate better with parents around common cultural dietary behaviors and practices in relation to evidence-based recommendations. Reaching through to parents when there were linguistic barriers was described as challenging. Furthermore, recommendations and information adapted to the Swedish way of eating and living caused additional communication challenges. Thus, having access to a translated evidence-based support tool, where parents had the opportunity to read through and process the information at their own pace, was described as a possible way to bridge certain cultural differences.

So [for parents] from other cultures, you live in a different way, and it will be very difficult if you have to fully adapt, as I think maybe you think differently about breakfast, lunch, dinner, snacks, that it might be harder for them to comply … because it’s a little different from how they live culturally. (Child healthcare nurse, 34 years, 5.5 years in the profession)

Furthermore, nurses also discussed how early introduction of sweets and sweet drinks was more common and acceptable in some cultures but also difficult to approach and influence during the short-scheduled visits at the child healthcare center. Nurses also underlined how socioeconomic vulnerability, which often conjoined with being new to the country, was a strong predictor and influencer of lifestyle behaviors. In line with this, parents also described how it was common and accepted in their culture to give children sweet treats and sweet drinks more frequently and from a young age and suggested that the app could be useful for increasing knowledge about this.

Because if I think about my children, that they weren’t allowed to eat anything sweet until they were a year old, even maybe a little longer. But if I were new to the country … or just arrived in Sweden, then I would accept the idea that “Yes, but it doesn’t matter that they get sweets already from six months … we have that in our culture, that you should … the children should get to taste everything to get used to it later. (Arabic/Swedish-speaking mother, 33 years)

### Support with future potential

The app was acknowledged as a preventive tool with potential for reaching diverse groups and purposes. It could be disseminated nationally through child healthcare to reach all parents but also to reach specific population groups that would benefit from additional support. From a health promotion perspective, the app had room for improvement in terms of functioning as a more long-term support for parents, starting from infancy with healthy food introduction.

#### A tool for diverse purposes and groups

Parents talked about the preventive potential of the app and how it could fill the need for reaching families early with supportive health behavior information on a population level. Dissemination of the app through child healthcare was viewed as important, relevant and logical as they reach most families. Child healthcare was also described as the most natural forum to turn to for support regarding questions on child nutrition and development, and parents acknowledged the app to be especially useful for first-time parents or for parents that in general felt unsure about healthy lifestyle behaviors.

I think that this [the app] is a good first effort to both identify and visualize certain patterns that often are quite easy to correct. (Swedish-speaking mother, 39 years)

Notably, parents highlighted that dissemination through child healthcare would further the use of the app, as most parents had high trust in them and a recommendation from child healthcare carried weight. Although some parents were open to also use the app if it was offered through other more commercial channels or forums, a connection with child healthcare was preferred.

I would prefer for the app to be offered through child healthcare, because that would catch my interest more, that ‘oh, maybe this is something very important for my child’ … it feels safer when offered through them. (Somali-speaking mother, 37 years (with interpreter))

The preventive potential of the app was further confirmed by the nurses who discussed the benefits of having an evidence-based digital support tool as a complement to their daily practice. The content in the app was expressed as already in line with the message that primary child healthcare strives to convey and that it would thus be an advantage both for parents and nurses in the long run if everyone gained access to the app. Furthermore, nurses acknowledged the need for supportive information about healthy eating behaviors as many parents worried about this and the app would be an appropriate support to offer parents in addition to the conversations about healthy dietary behaviors during the visits. However, the app would not replace the nurses’ conversation; instead, it would be something to use at home for deepened knowledge and insight. Both nurses and parents viewed the information in the app as equivalent to the information in the health conversations, albeit more comprehensive and accessible when needed.

It’s almost like we would stand behind them [the parents] a little bit every day for a period, I think, by getting the app. Because it’s hard to absorb everything we [child healthcare nurses] say when they’re here for a short visit and the kids are all over the place. So, I absolutely think that it [the app] can be a good complement, because it is evident that you can’t change anything after a short conversation. (Child healthcare nurse, 38 years, 12 years in the profession)

Apart from prevention, suggestions for expanded use of the app as part of child obesity treatment programs were expressed among both groups of informants. However, nurses also discussed the organizational challenges of using the app for treatment purposes, as that would require additional education, time and resources. Regardless, the opportunity for follow-up of health behaviors and body mass index (BMI) by using the app as common ground for conversation, feedback and advice during visits was highlighted as beneficial from a treatment perspective. The need for a practical and accessible digital tool to offer families where the child had overweight or obesity was also underlined due to the perceived lack of such support tools. In this case, the registration feature was highlighted as potentially beneficial. Registration could also provide opportunity for the nurses to follow the family's entries through the caretaker interface and, based on that, target and support the family's current needs.

You could then use it as a tool to say ‘download this app, and then you use it for a couple of months, and then we'll meet. And then we can look together at what changes you have made and what it has led to’ and so on. (Child healthcare nurse, 56 years, 2.5 years in the profession)

It would be good [registration linked to child healthcare center] because then the nurse can see how the child is eating. It would be beneficial for the child’s health. (Somali-speaking mother, 37 years (with interpreter))

Both informant groups described the accessibility of the app in different languages as a valuable addition to primary child healthcare; speaking another language should not be a barrier for accessing information available to everyone else. Furthermore, the increased future possibilities of reaching parents in need of an interpreter during the visits were brought up as especially valuable by the nurses, as informing about healthy lifestyle behaviors takes a longer time when a third party is involved. Most importantly, the app included a feature which enabled parents with limited literacy to also access the information content in the app through videos. Parents also expressed appreciation for this feature and shared how they had preferred watching the content in the app instead of reading it, as that was difficult for them. Even so, nurses also described a large interest in using the Swedish language version of the app among migrant parents that had begun learning Swedish. Thus, having both options was helpful.

#### Room for improvement

Suggestions on how the health promotion potential of the app and its features could be improved were expressed among both groups of informants. Parents expressed a strong interest in using an app that would follow their child's development throughout childhood and not be limited to the preschool years. Additionally, both parents and nurses discussed the potential benefits of using/offering the app already from birth, as a support for healthy food introduction. Furthermore, different ages were associated with different challenges, and thus, there was a need for age-relevant and supportive information for all developmental stages during childhood. Parents discussed how older children were naturally more open to impressions from peers and the outside world in general and how that posed additional challenges when setting boundaries, for example, for healthy diet and screen time behaviors. Therefore, access to information and strategies on how to maintain healthy habits as the child grew older were important.

Most three-year-olds are quite intense and energetic, but then you enter another [phase]. When the children are five, six years old, maybe there will be an increased inactivity, and above all an increased availability of screens for example. There might also be a need to boost and get a little more [support] on how to handle these habits. (Swedish-speaking mother, 39 years)

Some parents expressed a need for access to the app in its present form, for a longer period than the current 6 months, as the time needed for behavior change was individual. Having supportive information and tips to go back to for inspiration was considered helpful, and parents requested being able to visit the app freely from time to time when needed. The opportunity of continued use of the app could also result in a different, more relaxed use, when knowing how features like the registration worked and that it was available for a longer time.

It would be great for us to know … well, that you can keep it, that it doesn’t have to be for a specific period. (Somali-speaking mother, 22 years (with interpreter))

Another suggestion for improvement was making the app even more interactive to keep parents interested and motivated throughout the intervention period. Thus, although the regular content updates every 2 weeks was expressed as enough among some parents, others requested more frequent updates. Parents described that if they felt like they gained something new every time they entered the app, for instance, a recipe for a healthy snack or meal, it would most likely increase the chances of them engaging further with the information and features in the app. Moreover, although parents appreciated the parenting advice and strategies already available in the app, these were also described as focused on diet and activity behaviors. As children's psychosocial development was perceived as equally important but also as strongly linked to children's physical health, inclusion of parental strategies in general was suggested. Overall, parents were curious and expressed a need for more information and support regarding parenting and parenting styles in general, including how to be with your child as well as how to be more involved and promote your child's emotional and psychosocial development at different ages.

You can add more about the emotional and psychological things concerning the health of a child. This would be very helpful, because many parents also struggle with how to connect with their children, and how to behave with their children. (Arabic-/English-speaking mother, 28 years)

## Discussion

### Main findings

The findings in this qualitative study indicate that the MINISTOP 2.0 app was feasible, trustworthy, well accepted and appreciated as it increased knowledge and awareness around current health behaviors. Due to the accessibility in different languages and the possibility of disseminating the app at scale, both nurses and parents also described the app as an appropriate tool for reaching larger populations of parents as well as parents in need of additional support.

### Comparison with previous literature

One of the major findings in this study was the advantage of parents receiving relevant and evidence-based information on health behaviors for children in the preschool age that also was easily accessible and gathered in one place (within the app). Additionally, nurses being able to recommend and disseminate a digital support tool for parents, to engage with between visits, was also described as beneficial from a preventive point of view. When properly adapted, digital support tools such as the MINISTOP app may enable increased reach of migrant and other vulnerable population groups^18^, such as for example those with lower levels of literacy and/or health literacy. Development and evaluation of mHealth tools for implementation within healthcare settings is also in agreement with guidelines from the Swedish government and WHO,^13–15,33^ where healthcare systems are encouraged to incorporate digital healthcare services, accessible to the general population, not only to increase healthcare quality but also to decrease health inequities through increased accessibility to healthcare services.

Another relevant finding was the importance of being able to trust the source of the information to make lifestyle changes. This is also in line with findings from a qualitative study by Byambasuren et al.,^34^ who investigated barriers and facilitators for use of mHealth apps within healthcare. From the healthcare professional side, access to, for example, a list of apps from a trustworthy source to recommend to patients was considered a facilitator.^34^ Patients also expressed that being recommended or prescribed health apps by their physician was beneficial, as it made the process of finding an app from a trusted source less challenging.^34^ Moreover, both nurses and parents in the present study acknowledged the preventive potential of the app if disseminated through primary child healthcare, which further strengthens the importance of healthcare systems implementing and disseminating evidence-based digital support tools^13–15^ for self-management of, for example, health behaviors. Besides prevention, nurses also suggested a more targeted use of the app within child obesity treatment programs by using the caregiver interface connected to the app. Although this could be an additional way of using the app in the future, it should be noted that this would require additional resources. Nevertheless, based on the findings, the app is perceived as a feasible support to offer parents as part of routine child healthcare.

Noteworthy, parents described an increased awareness regarding their child's health behaviors after using the registration feature, as it visualized both time in active play and screen time as well as intakes of key dietary items over time. In a qualitative study of user perceptions of mobile health apps by Peng et al.,^35^ informants also described gaining increased awareness from tracking their health behaviors, especially when these were also presented graphically, as this facilitated interpretation over time. Indeed, using tracking or self-monitoring of health behaviors is a common behavior change technique used in health behavior change interventions^36^; however, literature also indicates a higher level of complexity in regard to health behavior change, where a combination of many interacting behavior change techniques may be more efficient than focusing on one specific technique alone.^37^ Findings from the present study also support this, as parents shared different experiences of what they found helpful in the app; some preferred registration alone or a combination of registration and information, while others perceived registration as stressful and instead preferred reading or watching information.

Both informant groups brought up cultural differences and their implications for health behaviors or when communicating about health behaviors. However, it is important to emphasize that the primary purpose of this paper was not to identify, discuss or compare cultural differences in health behaviors. Instead, we wanted to gather the voices of parents, including both Swedish- and foreign-born parents representing the current largest migrant populations in the country, to improve the app. Thus, this paper contributes information regarding what parents and nurses found supportive and important in an app-based intervention and may be used as a guide when developing similar digital interventions for parents. This is highly important, in order to be able to offer accessible digital supportive tools for healthy lifestyle behaviors for all families in Sweden.

#### Strengths and limitations

A strength of this study was the purposive sampling of parents via health communicators that enabled reach and recruitment of parents with limited knowledge in Swedish, as well as parents with limited literacy. Another strength was that the nurses represented various geographic and socioeconomic areas in Sweden which also contributed to diverse experiences of promoting healthy lifestyle behaviors to parents. One limitation might be that all nurses and parents were female, except for one father, and therefore, we do not know if the findings would have been different if more men had participated. Using a translator may also be considered a limitation; however, it was necessary for exploring user experiences of parents with limited literacy and/or knowledge in Swedish. With that said, the risk of information not being completely or correctly retold when using interpreters should always be considered; however, findings from these interviews were reasonable, and in line with findings from the other interviews, thus, we have no reason to question their credibility. Conducting phone interviews instead of face-to-face interviews could also be viewed as a limitation; nevertheless, experiences from previous studies indicate that there is little difference when comparing data collected over the phone vs. face-to-face.^38^ Additionally, as all interviews were conducted during the COVID-19 pandemic, interviews over the phone were the most suitable option.

Measures to increase the trustworthiness^32,39^ of the study and the findings through a credibility aspect included the recruitment of a diverse group of informants; parents had different cultural backgrounds and thus were able to share many different types of experiences. Moreover, participant triangulation by inclusion of informants on two different levels (parents and child healthcare nurses) provided different but interrelated experiences answering to the aim. Credibility was further endorsed by investigator triangulation, where data was analyzed and processed by two different researchers (C.A. and L.J.). Additionally, the interpretation of data was regularly discussed with two other researchers experienced in qualitative methodology (S.R. and A-K.L.). Finally, inclusion of quotations further strengthened the credibility of the findings, by increasing transparency. A description of the participant characteristics, the intervention and intervention setting were included to facilitate transferability of the findings. Dependability was ensured by following a pre-defined systematic data collection procedure for all interviews, as well as by using a semi-structured interview guide. Finally, the COREQ 32-item checklist^31^ was used to ensure inclusion of all necessary approaches to increase trustworthiness.

#### Implications

Overall, parents and child healthcare nurses reported high satisfaction with the MINISTOP 2.0 app and considered it a valuable tool for promoting healthy lifestyle behaviors in families with a 2-to-3-year-old child. These findings complement and support our previous quantitative evaluation of the trial where we reported significant positive effects on children's lifestyle behaviors and PSE.^18^ Noteworthy, parents also provided suggestions for several improvements for the app. For instance, although the information and recommendations in the themes of the app were appreciated, parents also asked for other types of content to be added, such as tips on activities, games, arts and crafts, as well as more recipes on healthy meals and snacks. Furthermore, parents suggested such content to be used for updating the app more frequently, as it would pique the parents’ interest and potentially increase the use of the app. Finally, parents brought up increased awareness in regards of portion sizes of food and amounts of sweet drinks and sweets after using the app and how it had affected them positively. Nevertheless, it also needs to be mentioned that although such practical tips are valuable and helpful, it is important to keep a balance by also informing parents that a child's hunger and satiety cues are individual and should be respected. Thus, the portion size pictures within the app are recommendations, or averages, meaning that some children will eat a bit more and some less. All these suggestions are considered for the 3.0 version of the MINISTOP app where we also will evaluate cost-effectiveness.

#### Conclusions

Overall, the results show that the MINISTOP 2.0 app was well received by both parents and healthcare professionals. Parents reported that the app increased their knowledge and awareness of healthy diet and activity behaviors for children and that it was a particular strength that it was recommended through child healthcare. Nurses perceived the app as a useful tool in their work with families on healthy lifestyle behaviors and obesity prevention. The accessibility in different languages was highly appreciated by both groups. These findings provide further evidence for a large-scale implementation of the MINISTOP app within Swedish primary child healthcare.

## Supplemental Material

sj-docx-1-dhj-10.1177_20552076231203630 - Supplemental material for User experiences of an app-based mHealth intervention (MINISTOP 2.0) integrated in Swedish primary child healthcare among Swedish-, Somali- and Arabic-speaking parents and child healthcare nurses: A qualitative study Click here for additional data file. Supplemental material, sj-docx-1-dhj-10.1177_20552076231203630 for User experiences of an app-based mHealth intervention (MINISTOP 2.0) integrated in Swedish primary child healthcare among Swedish-, Somali- and Arabic-speaking parents and child healthcare nurses: A qualitative study by Christina Alexandrou, Stina Rutberg, Linnea Johansson and Anna-Karin Lindqvist, Ulrika Müssener, Marie Löf in DIGITAL HEALTH

sj-docx-2-dhj-10.1177_20552076231203630 - Supplemental material for User experiences of an app-based mHealth intervention (MINISTOP 2.0) integrated in Swedish primary child healthcare among Swedish-, Somali- and Arabic-speaking parents and child healthcare nurses: A qualitative study Click here for additional data file. Supplemental material, sj-docx-2-dhj-10.1177_20552076231203630 for User experiences of an app-based mHealth intervention (MINISTOP 2.0) integrated in Swedish primary child healthcare among Swedish-, Somali- and Arabic-speaking parents and child healthcare nurses: A qualitative study by Christina Alexandrou, Stina Rutberg, Linnea Johansson and Anna-Karin Lindqvist, Ulrika Müssener, Marie Löf in DIGITAL HEALTH
