# Supplementary material for: User experiences of an app-based mHealth intervention (MINISTOP 2.0) integrated in Swedish primary child healthcare among Swedish-, Somali- and Arabic-speaking parents and child healthcare nurses: A qualitative study
Source: Digit Health. 2023 Sep 24;9:20552076231203630. doi: 10.1177/20552076231203630 (PMC10521279; doi:10.1177/20552076231203630)
Supplement: sj-docx-1-dhj-10.1177_20552076231203630 - Supplemental material for User experiences of an app-based mHealth intervention (MINISTOP 2.0) integrated in Swedish primary child healthcare among Swedish-, Somali- and Arabic-speaking parents and child healthcare nurses: A qualitative study [file sj-docx-1-dhj-10.1177_20552076231203630.docx]

**Supplementary file 1. Interview guides**

Alexandrou *et al.* User experiences of an app-based mHealth intervention (MINISTOP 2.0) integrated in Swedish primary child health care among Swedish-, Somali-, and Arabic-speaking parents and child health care nurses: a qualitative study

**Parents**

*Overall/general questions about the MINISTOP 2.0 app:*

What was your overall experience of using the app?

What was helpful/interesting to you?

Has the app affected your and your family’s/children’s diet and physical activity behaviors?

What type of support do you need in an app for healthy lifestyle behaviors?

Did the app provide that?

Think about the different features in the app. What are your thoughts in terms of their user friendliness?

What are your thoughts about the layout and design of the app?

What are your thoughts on the quality of the translated content in the app?

Was the language easy to understand?

*Specific/in-depth questions about the content and features in the app:*

What are your thoughts about being offered the app through primary child health care?

Were the instructions that you received from your child health care center, on how to get started and use the app, adequate?

What are your thoughts about the length of the time period (six months) that you were able to use the app?

What are your thoughts regarding the timing (during the 2.5-/3-year routine visit to primary child health care) that you were offered the app?

What did you think about the content in the app? (theme texts, library, weekly menus, videos).

What are your thoughts about a new theme being introduced every 14th day?

Would you have preferred more app content available in video or audio format?

Which features in the app did you perceive as most useful?

If you used the registration feature, which categories did you register in/use?

When using the registration feature in the app, you were able to view and follow your registrations graphically, over time. What did you think about these graphical outputs?

How were you affected by the feedback you received in the app regarding the registrations of your child’s diet, physical activity, and screen time?

The app also sent out push-notifications with reminders and general information, tips, and strategies on healthy lifestyle behaviors. What are your thoughts of these messages?

Do you remember any of the messages/push-notifications, was there any that made a special impression on you?

*Questions on overall app-usage and other similar apps:*

We are curious if your use of the app has changed during these six months that you were able to use it. How did you use the app during this period?

Is there anything that would have made you use the app more?

Have you used any other apps about your child’s health, diet, and physical activity behaviors?

Would you recommend others to use the app?

If you think freely: what would a perfect app to support parents in promoting healthy diet and physical activity behaviors in preschool-aged children look like?

**Nurses**

Can you tell me about your professional role and work?

How does your organization currently work with mHealth?

Can you tell me how you went about recruiting families to the study?

What challenges have you and your colleagues encountered during the recruitment?

What has motivated you and your colleagues to recruit families to the study?

Can you tell me about the feeling within the group during the MINISTOP 2.0 trial?

How have you and your colleagues used the MINISTOP 2.0 app in your daily work?

What do you think the parents' interest in downloading and using the MINISTOP 2.0 app will be like, when it is not part of a study?

What are your thoughts regarding the content in the app?

What are your thoughts of the caregiver interface?

Would you have changed anything in the app/caregiver interface?

What would you and your colleagues need to enable you to implement mHealth such as the MINISTOP 2.0 app?

Please share your thoughts about whether or not [name of the organization] has what is required to implement mHealth.

Please share your thoughts if there are any special considerations prior to implementing mHealth, compared to other digital interventions.

Please share your thoughts about the future and the use of mHealth, such as the MINISTOP 2.0 app.
